# Measurement and analysis of inequality of opportunity in access of maternal and child health care in Togo

**DOI:** 10.1186/s12913-017-2647-8

**Published:** 2017-12-04

**Authors:** Yacobou Sanoussi

**Affiliations:** University of Lomé, Faculty of Economics and Management (FaSEG), Lomé, Togo

**Keywords:** Inequality of health opportunities, Human opportunity index, Dissimilarity index, Maternal and child health, Togo

## Abstract

**Background:**

Access to maternal and child health care in low- and middle-income countries such as Togo is characterized by significant inequalities. Most studies in the Togolese context have examined the total inequality of health and the determinants of individuals’ health. Few empirical studies in Togo have focused on inequalities of opportunity in maternal and child health. To fill this gap, we estimated changes in inequality of opportunity in access to maternal and child health services between 1998 and 2013 using data from Togo Demographic and Health Surveys (DHS).

**Method:**

We computed the Human Opportunity Index (HOI)—a measure of how individual, household, and geographic characteristics like sex and place of residence can affect individuals’ access to services or goods that should be universal—using five indicators of access to healthcare and one composite indicator of access to adequate care for children. The five indicators of access were: birth in a public or private health facility; whether the child had received any vaccinations; access to prenatal care; prenatal care given by qualified staff; and having at least four antenatal visits. We then examined differences across the two years.

**Results:**

Between 1998 and 2013, inequality of opportunities decreased for four out of six indicators. However, inequalities increased in access to antenatal care provided by qualified staff (5.9% to 12.5%) and access to adequate care (27.7% to 28.6%).

**Conclusions:**

Although inequality of opportunities reduced between 1998 and 2013 for some of the key maternal and child health indicators, the average coverage and access rates underscore the need for sustained efforts to ensure equitable access to primary health care for mothers and children.

## Background

Access to maternal and child health services is critical for human development [1–3]. Investments to improve the quality of care received by mothers during and after pregnancy have contributed to significant reductions in maternal mortality rates. For example, the global maternal mortality rates declined from 385 maternal deaths per 100,000 live births in the 1990s to 216 maternal deaths per 100,000 live births in 2015 [4]. The decline in the global maternal mortality rate has mirrored a similar decline in under-five mortality rate. It is estimated that the lives of 48 million children younger than five years of age were saved between 2000 and 2015 [5]. Despite these improvements in maternal and child health indicators, infectious diseases and neonatal complications, which are readily preventable or treatable [5], remain responsible for the vast majority of under-five deaths and significant inequalities remain in access to maternal and child health services globally [6, 7].

The situation in Togo largely mirrors global trends. For example, infant mortality declined from 90 deaths per 1000 live births in the 1990 to 52 deaths per 1000 lives birth in the 2015 period [5]. The under-five mortality declined from 23 deaths per 1000 lives birth to 20 deaths per 1000 lives birth over the same period [5]. Similarly, maternal mortality declined from 553 deaths per 100,000 live births over the 1991–1998 period to 355 deaths per 100,000 live births over the 2008–2014 period [8].

Like many other low and middle income countries, the Togolese government has shown commitment to improving maternal and child health and reducing inequalities in health care access. For example, the 2012–2015 National Health Development Plan aimed to reduce maternal, neonatal, infant and child mortality and to facilitate universal access to essential health services. Despite these efforts, recent surveys in Togo show that inequalities persist. For example, households living in rural areas and those from poorer households have lower access to health services than those in urban areas or from wealthier households [9]. Further, under-five mortality rates are considerably lower in urban areas (69 deaths per 1000 lives birth) than in rural areas (106 deaths per 1000 lives birth) [10].

Inequalities in access to health care have sparked interest in understanding their drivers to inform interventions. However, empirical studies in Togo have primarily examined the total inequality of health and the determinants of individuals’ state of health [11–14]. One unanswered question, therefore, is whether these inequalities are associated with the supply of or demand for health care. To address this gap, we assessed the extent to which inequalities are associated with demand side socio-economic factors in order to understand the extent to which individuals have access to the available supply of health care.

### Measuring health inequalities: An overview of literature

Health inequalities can be evaluated using univariate [15] or opportunities approaches [16–19]. Using the univariate approach, inequalities are analyzed by examining differences in individuals’ health in a given population. In this approach, health is conceived as an intrinsic component of well-being and is analyzed without reference to inequalities in other dimensions of well-being [20]. Several studies have been based on this approach [21–23]. However, the univariate approach can be considered restrictive because it precludes analyses to examine different correlates of health inequalities [24].

In contrast, the opportunity approach, accounts for differences in the correlates of inequalities and takes into account individual’s life circumstances (individual, household, and/or geographic characteristics outside individual’s control that can affect access) and efforts (causes for which individuals are responsible) [25]. It is this distinction that guides the opportunity approach, whose results are better suited to inform the implementation of targeted policies to reduce inequalities.

One of the tools used to measure inequality of opportunity is the Human Opportunity Index (HOI). The HOI is a measure of the coverage rate of an opportunity, discounted by inequality in its distribution across life circumstances or circumstances groups (sets of individual with the same set of circumstances) [26]. The coverage is the percentage of individuals that have access to the opportunity. The HOI ranges from 0 (high inequality) to 100 (universal access). The HOI enables the measurement of the marginal contribution of each circumstance to inequality of opportunities.

Studies on inequalities of opportunity have focused on high income countries and analyzed the triangular relationship between childhood life circumstances, individuals’ efforts and their state of health. These studies often use cohort data, which are rare in many low and middle-income countries. In contrast, studies on the inequality of opportunities in low- and middle-income countries tend to rely on cross-sectional surveys such as Demographic and Health Surveys (DHS) [27–30].

In this study, we used nationally-representative data from the 1998 and 2013 Togo DHS (https://dhsprogram.com/data/available-datasets.cfm) to estimate inequality of opportunities in children’s access to primary care by evaluating mothers’ access to prenatal and postnatal care. Specifically, our objectives were threefold: (i) to estimate the coverage (prevalence) of access to care and the share of inequality of opportunity taking into account individuals’ circumstances; (ii) to identify which inequality of opportunity and average coverage rates are associated with observed access rate variations; and (iii) to estimate the relative contribution of individual, household, and geographic characteristics (circumstances) to unequal opportunities.

## Methods

### Data source

DHS data are collected using three types of questionnaires: a household questionnaire, which collects information on all members of the household as well as household characteristics; an individual woman questionnaire that is administered to all women aged 15 to 49 years in sampled households; and a male questionnaire that is administered to all men aged 15 to 59 years in the same households. The woman’s questionnaire includes a children’s health section. For the 1998 DHS, data were collected on 7517 households, 8569 women; 3819 men, and 3873 children under five. For the 2013 DHS, data were collected on 9549 households; 9480 women; 4476 men and 6535 children.

### Indicators

For each child born in the five years preceding the survey, mothers reported on child health outcomes as well as their utilization of health care services. We considered the following indicators: access to prenatal and postnatal care, as well as immunization. Access to prenatal care was assessed using two indicators: four or more antenatal visits; and receipt of the following during the visits: vital signs measurement, as well as blood and urine tests. To assess postnatal care, we considered whether the births were attended by trained personnel and the places where the deliveries occurred. The places of birth listed were: public and private health facilities, home and others. We assessed skilled birth attendance as the proportion of births in the five years preceding the survey that occurred in public or private health facilities. We considered mothers of children born in public and private health facilities to have access to skilled birth attendance and good quality postnatal care. Because immunized children are less likely to contract certain vaccine preventable childhood illnesses, we also assessed whether or not children had received any vaccinations as a measure of access to care.

From these variables, we constructed a composite indicator of access to adequate care for children. The composite indicator takes the value of 1 if all the following conditions are met: the child was born in a public or private health facility; the child had received any vaccinations; the child’s mother had access to prenatal care; the child’s mother received prenatal care from qualified staff; and the child’s mother reported at least four antenatal consultation visits.

### Analyses

For each type of care (prenatal, postnatal, vaccinations), we computed the human opportunity index (HOI) and the dissimilarity index (D-index) as measures of inequality of opportunity. We drew on a methodological framework based on measures of inequality of opportunity used in previous studies [3, 31, 32]. Indicators of access to health care were considered as opportunities available in the children’s area of residence. The inequality of opportunity was measured by the D-index, which is the share of the number of opportunities that must be reallocated given the circumstances of life to ensure equality in access to these opportunities. We estimated the D-index using a three-step process:

In the first step, we estimated the conditional likelihoods by specifying a binary function between access to care and circumstance variables using a logistic or probit regression between children access to health care and several variables of circumstances (x_1_..x_2_.. x_3_….. Xn) that offer different opportunities.

In the second step, we estimated the predicted probability of access to care considering the circumstances variables chosen for each individual. In the final step, we estimated the probability of access to care $\overline{p}$ and the D-index using the equations below:

$$ {\displaystyle \begin{array}{l}\overline{p}={\sum}_{i=1}^n{w}_i{p}_i\;\mathrm{where}:{w}_i=\frac{1}{n}\;\mathrm{and}\;n\;\mathrm{is}\  \mathrm{the}\  \mathrm{sample}\  \mathrm{size}\\ {}D- index=\frac{1}{2\overline{p}}{\sum}_{i=1}^n{w}_i\left|p-\overline{p}\right|\end{array}} $$

*D* measures the dissimilarity between access of basic services for groups defined by circumstance characteristics and the average access rate for the same service for the population as a whole. It measures also the share of opportunities that must be reallocated given the circumstances of life to ensure equality in access to this opportunity. The *D*-index ranges from 0 to 1 (0 to 100 in percentage terms), and in a situation of perfect equality of opportunity, *D* will be zero.

#### Human opportunity index

Empirically, the HOI for basic services such as health represents the adjusted (for difference in access) coverage in the access of these basic services. The level of opportunity measured by this index (access rate) can be interpreted as the number of opportunities in a society that have been allocated based on an equal opportunity principle. The HOI was computed using the formula below:

$$ HOI=\overline{p}\left(1-D\right) $$

where *D* is the index of inequality of opportunity in the access of basic services for groups defined by circumstance characteristics compared with the average access rate for the same service for the population as a whole [3]. (1–*D*) is equal to 1 if the access to opportunity is independent of circumstances, in the situation where the HOI is equal to the average access ($\overline{p}$). It means that when the inequality of opportunity is zero, the coverage rate becomes equal to that of access to the opportunity.

Since we were performing a comparative analysis, we tested whether the *D*-index or the average access accounted for the overall change in the HOI between 1998 and 2003. Therefore, we decomposed the access rate in scale effect (variation in coverage ratio) and distribution effect (variation in unequal opportunities) [3]. Any change in the HOI can be attributed either to a variation in the coverage rate or a variation in the index of inequality of opportunity:

$$ {\displaystyle \begin{array}{l} Variation of\  HOI\kern2em {HOI}^{final}-{HOI}^{initial}=\varDelta \overline{p}+\varDelta D\\ {} Scale Effect\kern4em \varDelta \overline{p}={\overline{p}}^{final}\left(1-{D}^{initial}\right)-{\overline{p}}^{initial}\left(1-{D}^{initial}\right)\\ {} Distribution Effect\kern1.5em \varDelta D={\overline{p}}^{final}\left(1-{D}^{final}\right)-{\overline{p}}^{final}\left(1-{D}^{initial}\right)\end{array}} $$

### Decomposition of the dissimilarity index by the Shapley value

To capture the contribution of each circumstance variable *c*_*j*_to inequality opportunity, we estimated their marginal impacts using the Shapley value [27] using the formula:

$$ {D}_{c_j}=\sum {s}_{CN/\left({c}_j\right)}\frac{s!\left(n-s-1\right)!}{n!}\left[D\left(S\cup \left\{{c}_j\right\}\right)-D(S)\right] $$

Where:

*N*, the total of the circumstances.

*n*, number of selected circumstances in *N*.

*s*, a subset of *N* with s circumstances without ***c***_***j***_

*D*(*S*), the *D*-index estimated with *S*

*D*(*S* ∪ {*c*_*j*_}), the *D*-index calculated with the subset of circumstances **S** and the circumstance ***c***_***j***_

with (*N*), the *D* − *index* for all the circumstances variables retained, the contribution of ***c***_***j***_ to *D* − *index* is given by:

$$ {\theta}_{c_j}=\frac{D_{c_j}}{D(N)} $$

(with) $$ \sum {D}_{c_j}=1 $$

We used the IOP (Inequality of opportunity) module in Stata Version 12 [33].

## Results

The average coverage rates or prevalence, the inequality of opportunity and the HOI values by year are presented in Table 1. The average coverage rate declined between 1998 and 2013 for immunization (from 55.0 to 25.7) and for prenatal care provided by qualified staff (from 84.9 to 68.9). For the other three indicators and the composite, the average coverage rate increased between 1998 and 2013.

**Table 1:** Human Opportunity Index, coverage rate ($\overline{\mathrm{p}}\Big)$ and opportunity inequality (D)

Health Care Access Indicators	Average coverage rate or prevalence $\overline{p}$		Inequality of opportunity D		(1-D)^a^		Human Opportunity Index (access rate)	
	1998	2013	1998	2013	1998	2013	1998	2013
Birth in a public or private health center	46.08	71.37	24.81	14.31	75.19	85.69	34.65	61.16
Access to immunization	55.01	25.70	12.23	8.48	87.77	91.52	48.29	23.52
Access to prenatal care	82.31	92.77	5.97	3.03	94.03	96.97	77.39	89.96
Access to prenatal care given by qualified staff	84.87	68.88	5.86	12.49	94.14	87.51	79.90	60.27
Having at least four antenatal visits	46.06	54.25	13.12	11.65	86.88	88.35	40.02	47.92
Adequate care	6.53	9.30	27.74	28.64	72.26	71.36	4.72	6.62

^a^Difference between the unit (1) and index D

**Fig. 1:**
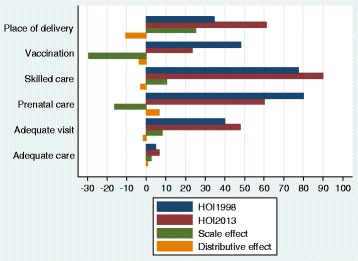
Decomposition of the HOI in scale and distribution effects

Figure 1 shows the variations in the access rate, which estimates the number of opportunities existing in a society and that are allocated on the basis of the principle of equal opportunity, for each opportunity indicator as well as the scale and distribution effects. Access to immunization and prenatal care given by qualified staff declined between 1998 and 2013. The reduction in the rate of access to immunization is explained by the combined effect of a decrease in the average coverage rate and inequality of opportunities. The scale effect explains 29.3% of the decrease of the access rate. The scale effect outweighed the distribution effect (which improved by 3.8%). Similarly the reduction in the rate of access to prenatal care provided by qualified staff is explained by a decrease in the average coverage rate and inequality of opportunities. For the other four indicators, with the exception of access to adequate care, the increases in access rates is explained by an increase in the average coverage rate and a decrease in opportunity inequality. However, the magnitude of the variation in the average coverage rate outweighed the inequality of opportunity. Thus, the scale effect appeared to be the main source of the increase in the rate of access to these opportunities.

The contribution of each variable of circumstances to the inequality opportunity is presented in Table 2. The most important contributors to inequality of opportunity were education of the parent (father and mother), region and residence. In 1998, the place of residence was the most important factor contributing to the inequality of access to birth in a qualified health center. However, its contribution decreased from 20.1 to 11.7 in 2013. In 2013, region was the most significant contributor to inequality of access to child vaccinations (59.8). The contribution of most circumstance variables decreased between the two years.

**Table 2:** Contribution of circumstances variables to indicators of access to maternal and child health care services

		Birth in a qualified center	Immunization	Access to prenatal care	Access to prenatal care given by qualified staff	Access to required consultation visits	Adequate care
Mother’s education	1998	18.58	20.71	22.09	19.54	21.37	15.91
	2013	15.5	7.57	28.2	7.38	18.1	7.03
Father’s education	1998	17.3	23.7	23.3	24.4	24.2	22.4
	2013	9.84	0.54	12.38	8.11	7.79	7.22
Mother’s occupation	1998	4.71	4.44	6.82	7.57	4.32	2.39
	2013	9.41	9.62	11.28	5.71	8.43	6.91
Father’s occupation	1998	16.45	20.49	17.84	18.15	15.88	8.57
	2013	16.07	3.77	9.19	14.74	13.28	14.1
Household’s socioeconomic status (wealth quintile)	1998	6.63	2.28	1.41	1.36	4.03	9.62
	2013	21.18	5.06	12.34	22.32	18.75	18.67
Sex of the head of household	1998	2.8	5.4	1.98	2.08	2.74	9.47
	2013	2.33	3.39	0.73	3.51	1.07	1.9
Children’s Sex	1998	0.25	0.61	1.64	1.72	0.71	0.25
	2013	0.13	6.42	0.1	0.32	0.71	3.16
Number of children in the household	1998	5.81	3.58	8.01	7.76	7.17	5.21
	2013	4.8	1.76	9.28	4.32	6.31	5.15
Region	1998	7.33	4.92	2.91	1.93	4.53	13.82
	2013	9.06	59.84	5.13	12.49	8.41	13.73
Residence	1998	20.13	13.83	14.05	15.53	15.07	12.37
	2013	11.74	2.02	11.39	21.1	17.43	22.12

## Discussion

We found that inequality of opportunity decreased for most of the opportunities between 1998 and 2013. Overall, the findings suggest that access to maternal and child health services has improved over time as indicated by the proportions of births in public or private health facilities, the proportion of immunized children, the proportion of mothers reporting at least four antenatal visits and the proportion of mothers reporting receipt of prenatal care. In terms of decomposition we found that, in general, the main source of variations in the access rate to different opportunities was the scale effect. We also observed a decrease in the contribution of most circumstances variables over the period considered.

The change over time in the average coverage and access rates is associated with variations in the inequality of opportunity. This finding suggests that efforts to decrease inequalities in access to healthcare have been successful. However, the inequality of opportunity is still considerable and suggests the need for sustained efforts to ensure equal access to opportunity.

To understand the evolution of access to opportunities over time, we investigated which of the two components (the inequality opportunity and the average coverage rate) was the main source of variations observed by decomposing the access rate in scale effect and distribution effect. The scale effect was measured by variation in coverage rate while distribution effect was measured by variation in inequality of opportunities. The decomposition analysis showed the extent of scale effect variations and changes over time. Our results suggest that the scale effect negatively affected the rate of access to health care. However, the contribution of inequality of opportunity through the distribution effect was not negligible. Thus, inequality of opportunity would have a negative influence on the rate of access to health care opportunities. Our results are consistent with those from earlier studies [27, 28] that have found that the scale effect is the main source of variations in the rate of access to different opportunities. This implies that one way to improve the access rate to different opportunities is to implement policies that increase coverage rate of health services.

In terms of circumstances, the parents’ (father and mother) education, region and residence were the most significant contributors. Previous studies have also shown that region and residence [2, 28, 30, 34] as well as parents’ education [34] have an important influence on health inequality. These results suggest that improvement in health indicators will require investments in other sectors such as education.

Study findings should be interpreted in light of the following limitations. First, although we compared the results from the 1998 and 2013 DHS, we were unable to compute trends. Given the 15-years duration between the surveys, our decomposition of effects analysis may not have adequately captured variation. Second, given the large time difference between the two observation periods other macro-economic policies and changes in the health environment may have occurred that could confound the findings. However, an examination of these broader changes was beyond the scope of the study.

## Conclusion

Our findings suggest that there have been significant improvements in the availability of and access to maternal and child health services in Togo. These improvements may explain the decreases observed in the inequality of opportunities and the concomitant increase in the average coverage and access rates to select maternal and child health services. However, levels of inequality of opportunities as well as the average coverage and access rates demonstrate that there are still significant gaps in ensuring equitable access to health care. These results point to the need for sustained efforts to reduce unequal opportunities in order to ensure equitable access to healthcare opportunities.

## Abbreviations

DHS: Demographic and Health Survey
HOI: Human Opportunity Index
IOP: Inequality of Opportunity
